# New Developments in Cathodoluminescence Spectroscopy for the Study of Luminescent Materials

**DOI:** 10.3390/ma10030312

**Published:** 2017-03-17

**Authors:** Daniel den Engelsen, George R. Fern, Paul G. Harris, Terry G. Ireland, Jack Silver

**Affiliations:** Centre for Phosphor and Display Materials, Wolfson Centre for Materials Processing, Brunel University London, Uxbridge, Middlesex UB8 3PH, UK; ddenengelsen@onsbrabantnet.nl (D.d.E.); george.fern@brunel.ac.uk (G.R.F.); paul.harris@brunel.ac.uk (P.G.H.); terry.ireland@brunel.ac.uk (T.G.I.)

**Keywords:** luminous efficacy, secondary electrons, backscattered electrons, charging, shielding, electron microscopy, decay, STEM-image, panchromatic image

## Abstract

Herein, we describe three advanced techniques for cathodoluminescence (CL) spectroscopy that have recently been developed in our laboratories. The first is a new method to accurately determine the CL-efficiency of thin layers of phosphor powders. When a wide band phosphor with a band gap (E_g_ > 5 eV) is bombarded with electrons, charging of the phosphor particles will occur, which eventually leads to erroneous results in the determination of the luminous efficacy. To overcome this problem of charging, a comparison method has been developed, which enables accurate measurement of the current density of the electron beam. The study of CL from phosphor specimens in a scanning electron microscope (SEM) is the second subject to be treated. A detailed description of a measuring method to determine the overall decay time of single phosphor crystals in a SEM without beam blanking is presented. The third technique is based on the unique combination of microscopy and spectrometry in the transmission electron microscope (TEM) of Brunel University London (UK). This combination enables the recording of CL-spectra of nanometre-sized specimens and determining spatial variations in CL emission across individual particles by superimposing the scanning TEM and CL-images.

## 1. Introduction

Cathodoluminescence (CL) was discovered by the pioneers of gas discharge and cathode ray tubes (CRT) in the second half of the 19th century. Besides the development of vacuum technology and cathodes for these devices, the understanding and availability of luminescent materials was key to the commercial introduction of CRTs and gas discharge lamps (TL) in the middle years of last century [1,2,3,4]. In the wake of these industrial applications, the need for adequate characterisation techniques for the luminescent materials arose. In the second half of the 20th century, CL-spectroscopy was developed; this in combination with electron microscopy became a popular technique for studying semiconductors [5,6], minerals [7] and phosphors [8]. The phosphors in CRTs and TL were applied as micrometre-sized powders that were deposited onto a glass substrate. The advantage of this technology was that the luminous efficacy, which was not very much dependent on the thickness of such a phosphor layer, was inherently large due to the absence of light trapping in powder layers. Nevertheless, luminous efficacy and energy efficiency of the phosphors have always been an issue in both CRTs and TL, due to the commercial needs to minimize operational costs and improve the luminance. The 1990s and the 2000s saw an upsurge in the number of publications on field emission displays (FEDs), as it was believed that these flat displays could be a successor to bulky CRTs. The publication of Uchida et al. [9] on the surface-conduction electron-emitter display (SED) described the basic design of Toshiba-Canon’s FED, which was the result of the last large industrial effort to introduce FEDs into the market [10,11].

Practical difficulties meant that the anode voltage in a FED could not be made larger than about 5 kV, which implied a rather low luminous efficiency of the phosphor screen [10,11]. This triggered the search for efficient so-called low-voltage phosphors [12,13,14], which started in the 1970s and is still on going [15]. Cathodoluminescence spectroscopy is an indispensable tool in this research area for measuring spectra and colour coordinates, and the determination of luminous and energy efficiency of the phosphors. The measurement of CL-spectra and the calculation of colour coordinates thereof are straightforward and well-known methods, which will not be described herein. However, the determination of the luminous efficacy is less obvious as can be concluded from the review of Shea [12] and the paper of Chubun et al. [13]: the spread of values reported by different authors for the luminous efficiency at low electron beam (e-beam) voltage and low current density was found to be rather large; in many cases, by more than a factor of 2. The reasons for this variation of the efficacy were presumably difficulties in measuring the current striking the sample due to charging of the phosphor particles and incomplete compensation for secondary electron (SE) emission upon electron bombardment. Although Chubun et al. applied a thin Al-film on top of the powder layer to prevent charging, they still found a large variation of the luminous efficiency in the tested phosphors. The difficulty in measuring CL from insulating phosphor layers at low e-beam energy is due to the fact that a top layer of aluminium to prevent charging of the phosphor grains cannot be applied, since it will absorb too many of the beam electrons. This charging is negative in the case of the SE-coefficient γ being <1, or positive in the case that γ > 1. For most materials at low beam voltages γ is less than one, and so the material charges negatively. As the voltage increases then so does the value of γ, and for many materials may become greater than one. This causes the surface to charge positively, which suppresses further secondary emission and the surface potential stabilizes at a small positive value. If the beam voltage is further increased, however, then γ decreases and for all materials eventually becomes negative and the surface potential destabilizes and the material charges negatively again [3]. The second cross-over normally occurs at beam voltages of 1–2 kV (depending on the materials and morphology), which is well below the voltages normally used in CL-spectroscopy and -microscopy. At voltages just above the second cross-over the effect of negative charging is increasing the value of γ, but this is not normally sufficient to prevent charge build up. In this case the thin surface layer may charge up until it reaches the dielectric breakdown threshold of the material and then discharges, before resuming charging. As a result, the surface potential fluctuates rapidly. This behaviour can sometimes be observed when insulating specimens are examined in an electron microscope with the secondary electron images flashing rapidly with the fluctuating surface potentials. The extent of potential build-up depends on the material, its γ value, and how well it is able leak away the charge before the beam returns. The breakdown threshold is relatively modest because of the small thickness of the particles and photoconductivity induced in the surface upon electron bombardment. Seager et al. [16] measured negative charging in insulating phosphor layers with Auger electron spectroscopy (AES). They also did not find large charging voltages, possibly due the fact that the AES e-beam induced some conductivity in the insulating phosphor particles. Cho et al. [17] also found negative charging of Y_2_O_3_:Eu^3+^ layers in a FED upon screen loading.

In this article we shall describe a new technique to measure the luminous efficacy (and the energy efficiency) of insulating phosphor layers upon excitation with an e-beam. We coined this technique “comparison method”, because we used a conductive plate or a thin layer of a conductive phosphor powder to accurately adjust the power density of the impinging e-beam [18,19].

In conventional scanning electron microscopes (SEM) that are equipped with a photomultiplier (PM) tube the panchromatic image of the (luminescent) specimen can reveal features that cannot be observed with secondary electron or backscattered detectors. This well-known technique also enables the determination of decay times without beam blanking by evaluating the greyscale of smeared-out features. In this way, we could determine decay rates from 2 µs up to 0.1 s, which covers a range of almost 5 decades [20]. The determination of the decay time of CL-phosphors by this method is the second subject to be dealt with in this article.

Finally, the combination of microscopy and spectrometry in a transmission electron microscope (TEM) will be presented [21,22,23]. The JEOL TEM of Brunel University London was, when it was delivered, a unique instrument being equipped with a Vulcan^TM^ CL detector of Gatan (Pleasanton, CA, USA) for imaging (panchromatic) and spectroscopic purposes. This combination of microscopic and spectroscopic techniques enabled the recording of CL-spectra of nanometre-sized specimens and determining spatial differences in one crystal by superimposing the STEM and light images. An example of our recent work on (Lu_1-*x*_Gd*_x_*)_2_O_2_S:Tb^3+^ phosphors will be presented.

## 2. Cathodoluminescence Techniques

### 2.1. Comparison Method

The comparison method deals with the determination of the energy efficiency and luminous efficacy of CL. Before describing the comparison method we shall briefly recall the definitions of energy efficiency and luminous efficacy. The energy efficiency η*_e_* of CL is defined as the ratio of the power density *P_r_* of the CL and the power density *P_e_* of the e-beam that hits the phosphor layer (expressed in %):

(1) $$\eta_{e}=\frac{100P_{r}}{P_{e}}$$

The power density of the e-beam is the product of beam voltage and current density at the sample. The adjustment of the current density for phosphor samples is the essence of the comparison method, to be discussed hereafter. For powder samples, the angular distribution of the emitted light intensity is assumed to be Lambertian, which means that the power density of the CL can be calculated according to

(2) $$P_{r}=\pi R$$

where *R* is the radiance expressed in W/(sr·cm^2^). The radiance of the emitted light is defined as:

(3) $$R=\int_{a}^{b}SR(\lambda )d\lambda$$

where SR(λ) is the spectral radiance (in W/(sr·cm^2^·nm)) as a function of the wavelength λ and the integration limits, *a* and *b*, are determined by the eye sensitivity curve for reasons of comparison with the luminance. The luminance *L* (in cd/m^2^) of the CL is defined as:

(4) $$L=\int_{a}^{b}SR(\lambda )V(\lambda )d\lambda$$

where *V*(λ) is the eye sensitivity curve. The luminous efficacy η*_l_* (in lm/W) for a Lambertian light distribution is given by

(5) $$\eta_{l}=\frac{\pi L}{P_{e}}$$

**Figure 1:**
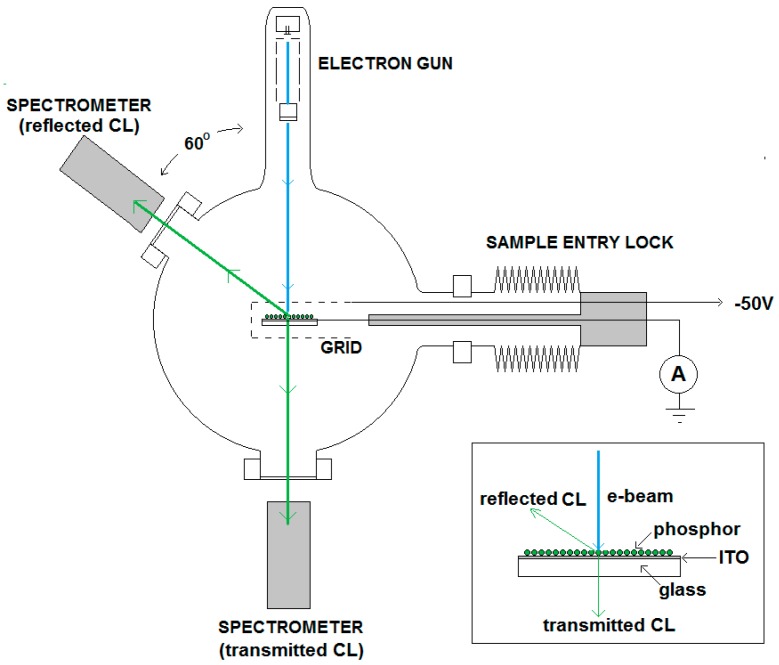
Spectrometers arrangement with vacuum chamber, electron gun, sample, shield, spectrometer 1 (reflection mode) and spectrometer 2 (transmission mode). In the 1–5 keV rig the sample is vertically oriented; in the 2–15 keV system the sample is oriented horizontally. The windows of the vacuum chamber are made of glass, which contains BaO to block X-rays at 15 keV. The insert represents a typical sample with phosphor layer deposited onto an ITO-film. The current is measured using a calibrated 1 MΩ resistor.

As mentioned above, the comparison method refers to an adjustment technique of the current density of an e-beam that is impinging on an insulating phosphor powder deposited as a thin film on a conductive substrate. The measuring set up for the samples has been depicted in Figure 1, while a detail of a typical sample has been represented in the insert. The substrate is a glass plate coated with a thin conductive, transparent film of indium tin oxide (ITO). In Figure 1, two principal directions for the emitted CL have been drawn: a reflection mode at the e-beam side and a transmission mode at the rear side of the glass plate. If the phosphor layer is very thick, the scattering events in the layer prevent transmission through the glass plate.

The coating thickness in our investigations was usually between 1 and 4 mg/cm^2^; this implied that for small phosphor particles with diameters <1 μm the light distribution of the CL in both modes is assumed to be Lambertian [18]. This assumption was explicitly made by Shea [12] and Shea and Walko [24], and implicitly by Chubun et al. [13]. For layers with more than three particles on top of each other, this assumption has been well established; for monolayers of particles. the intensity of the CL deviates from a pure cosine distribution. Since we measured the radiance (and luminance) of the CL in the phosphor layer at an angle of 30°, there could be a slight underestimation of the measured radiance (and luminance) for samples with a very thin coating thickness. The coating thickness of layers deposited by either settling or electrophoresis in our experiments was measured by weighing [18].

Figure 1 indicates that spectra are measured in both reflection and transmission mode. The advantage of this measuring method is that the sum of the radiances in the reflection and transmission modes is independent of the layer thickness for non-absorbing phosphor layers [18] as long as the energy of the e-beam has completely been transferred to the phosphor particles. The latter condition is not satisfied in very thin phosphor layers, which contain pinholes. The second characteristic of our measuring technique is the adjustment of the beam current onto a conductive sample, which does not charge upon electron bombardment. For conductive materials it is straightforward to determine the effective current impinging on the surface. The actual current refers to the primary electrons hitting the surface minus the SEs leaving from that surface. Thus, in order to determine the effective current for CL, the SEs must be sent back to the phosphor to be collected. This can be done by biasing the target surface positively, or using a shield or grid that is biased negatively. The latter method was chosen, because in this way the collection of SEs emitted from the wall of the vacuum chamber could be avoided. In most of our experiments the shield was at −50V. In the case of a charging sample, it is impossible to guarantee that all SEs are collected by biasing the shield negatively. In that case a spurious current will be measured. In order to deal with this problem, a comparison method has been developed: CL from a charging sample is measured by applying the same current settings as used for a non-charging reference surface such as Cu, ITO or ZnO:Zn. Copper and ITO are chosen because they have almost the same backscattering yields as ZnO:Zn. The additional advantage of using ZnO:Zn as non-charging reference is that the CL of ZnO:Zn can be used to optimally align the spectrometers 1 and 2.

The reference and sample are positioned in the e-beam by a vertical translation (1–5 keV rig) or horizontal translation (2–15 keV rig) as indicated in Figure 1. When making measurements, the reference is first positioned in the e-beam, the current is usually adjusted to 1 μA (as indicated by the high impedance ammeter), yielding a current density of 1 μA/cm^2^, since the surface areas of references and samples are typically 1 cm^2^. When the shield is biased to −50 V, low energy SEs from the reference sample are collected together with the primary electrons and thus a true measurement of the current striking the sample is made. The shield structure is designed to have as high a transparency as possible (about 98%) so that high energy backscattered electrons (BSEs) are able to escape unhindered: i.e., these are not detected in this way. BSEs do not contribute to the luminescence and must be discarded. It is worth noting that measurements of beam current made using enclosed Faraday cups normally collect these backscattered electrons, and so substantially over-estimate the energy absorbed by the phosphor. The conductive reference is ideally chosen to have a backscattering coefficient as close as possible to that of the sample, hence similar mean atomic number and morphology, so that the fraction of electrons backscattered from both is the same.

The sample is maintained at earth potential and so the kinetic energy of the electrons is proportional to the applied cathode voltage. This is not always the case in other studies; for example, in studies by Shea [12,24] and Wakefield et al. [25], the samples were biased positively, and therefore a correction must be made to calculate the kinetic energy of the primary electrons. In making CL measurements from charging and non-charging phosphor samples, the current adjustment as determined for the reference is not altered. The ITO-layer is connected to earth during the CL-measurements, but the current that may have changed in the case of a charging sample is not recorded. The assumption is that the same quantity of primary electrons hit the charging phosphor layer as determined for the non-charging reference. Obviously, this is only correct if the voltage of the charged phosphor surface does not deviate much from the voltage of the conductive reference. The work of Seager et al. [16] provides evidence for this hypothesis and by recording the so-called SE-yield curves we found that we could usually comply with this condition. This will be discussed hereafter.

**Figure 2:**
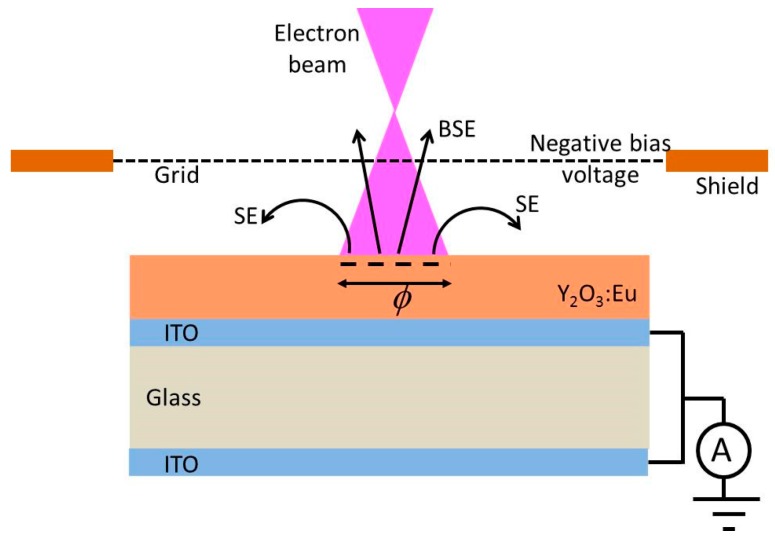
Over-focused e-beam hits Y_2_O_3_:Eu^3+^ phosphor layer and generates negative charge. φ is the diameter of spot. BSEs pass the grid (at −50 V), whereas SEs are deflected. Not all SEs are collected in the case of charging.

The size and luminance uniformity of the spot on the reference is visually optimized by adjusting the focus voltage of the electron gun. The focus voltage is about ~45% of the beam voltage in measuring the CL. At this focus voltage, the focus point of the electron gun is in front of the sample (over focus condition), as shown in Figure 2.

**Figure 3:**
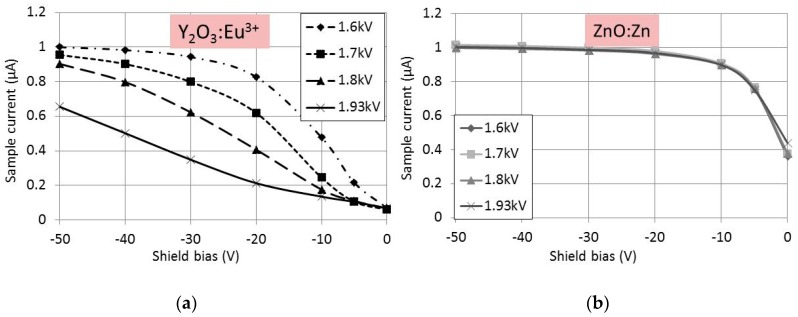
SE-yield curves at 3 keV primary beam energy and various focus voltages. Best focus is at 1.93 kV. (**a**) 2 mg/cm^2^ Y_2_O_3_:Eu^3+^; and (**b**) 2.2 mg/cm^2^ ZnO:Zn.

In Figure 2 the BSEs pass the negatively biased grid, whereas the slow SEs are deflected. However, in the case of charging, not all SEs will be collected, because some are repulsed by the negatively charged phosphor surface. By changing the focus voltage the size of the spot on the phosphor layer changed, implying that the the current density in the spot varies as well. The current density of the e-beam is, besides the voltage, the most important parameter to affect the charging of an insulating phosphor layer. This is shown in Figure 3, where the sample current has been plotted as a function of V_shield_. The curves in Figure 3 are called SE-yield curves.

In Figure 3 the SE-yield curves at various focus voltages for Y_2_O_3_:Eu^3+^ and ZnO:Zn have been plotted at a primary beam energy of 3 keV. The sample current was adjusted to 1 μA at V_shield_ = −50 V and a focus voltage of 1.6 kV. The effect of focus voltage was substantial for Y_2_O_3_:Eu^3+^, as seen in Figure 3a, whereas the SE-yield in the case of ZnO:Zn was independent of the focus voltage. At the best focus voltage of 1.93 kV, the diameter of the spot size on the sample was 2.5 mm. The spot size increased to 9 mm at a focus voltage of 1.6 kV. From the curves in Figure 3, we conclude that Y_2_O_3_:Eu^3+^ is charging negatively. The absolute value of the charge increased when the spot sizes decreased, i.e., when the current density increased. Figure 3a indicates that at high current density the shield voltage had to be decreased to collect all SEs. In some cases we had to decrease the shield voltage to −100 V to ensure complete collection. This proves that the charging voltages of Y_2_O_3_:Eu^3+^ and Y_2_O_2_S:Eu^3+^ phosphor layers are indeed limited, as described above.

**Figure 4:**
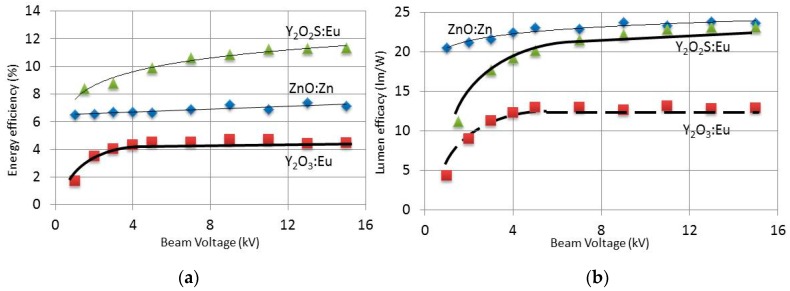
Cl efficiency of ZnO:Zn, nanosized Y_2_O_3_:Eu^3+^ and Y_2_O_2_S:Eu^3+^ as function of beam voltage at shield bias –50 V and current density of 1 μA/cm^2^: (**a**) energy efficiency; and (**b**) luminous efficacy.

Figure 4a,b shows the energy efficiency and luminous efficacy, respectively, of ZnO:Zn, Y_2_O_3_:Eu^3+^ and Y_2_O_2_S:Eu^3+^ as a function of e-beam voltage. These results were presented, discussed and compared with literature data previously [19]. We will suffice in making some comments on these results.

Figure 4a shows that the energy efficiency of Y_2_O_2_S:Eu^3+^ is the highest, whereas ZnO:Zn has the highest luminous efficacy (Figure 4b). The reason for this difference is due to cutting off the 707 nm emission peak of Y_2_O_2_S:Eu^3+^ by convoluting the spectrum and the eye sensitivity curve in measuring luminance, which is the physical quantity for determining luminous efficacy. Another interesting result indicated in Figure 4 is the levelling off of the efficiency curves for Y_2_O_3_:Eu^3+^, whereas the curves of ZnO:Zn and Y_2_O_2_S:Eu^3+^ show a monotonic increase.

The Y_2_O_3_:Eu^3+^ particles in this experiment were nanometre-sized with an average diameter of 300 nm (range of 100–500 nm), while the other two materials were micrometre-sized [19]. In the case of large monocrystalline phosphor particles without any defects, one expects that the CL efficiency would be proportional with V^n^, where V is the beam voltage and the exponent n is about 3.5. This follows from the fact that the penetration depth is proportional with V^1.66^ and the electron penetration volume is more or less spherical [20,26]. At low beam voltages the efficiency rises steeply in some cases; however, as soon as the penetration depth approaches the particle size, electrons can escape from the first particle and may enter a second particle. This is an inefficient process since it also creates SEs and BSEs. Moreover, the photons suffer multi-scattering events before escaping from the layer, which decreases the light output because the absorption coefficient in the layers is not exactly zero. These losses are more pronounced in the layers of the nanometre-sized Y_2_O_3_:Eu^3+^ than in the layers of Y_2_O_2_S:Eu^3+^ and ZnO:Zn and may explain the stronger levelling off for Y_2_O_3_:Eu^3+^. An alternative explanation is that there may be a thin surface dead layer on these materials, due for example to contaminant pick-up or due to slow back reaction with the atmosphere to more stable phases. For the coarse powders this dead layer contributes less to overall performance as the beam voltage (and therefore penetration) increases. This is not observed for the nanosized-Y_2_O_3_:Eu^3+^, because its particle size is so small that even at low voltages the beam penetrates all the way through the particles.

The comparison method enables a reliable determination of the CL efficiency of charging phosphor layers, because of the adjustment of the current density with a non-charging target. Another feature of the measuring technique is the evaluation of the radiance (and or luminance) in both reflection and transmissions modes, which means that the method is indifferent to variation of the layer thickness. The comparison method was devised to study the behaviour of double layers of phosphor particles for FEDs to enhance the luminance. The idea was to increase the light output of a phosphor screen by depositing a high-voltage phosphor on top of a low-voltage phosphor. Double and triple layers of phosphor particles were successfully used in so-called Penetrons to alter the CL-colour upon changing the beam voltage [27]. Mixing of the phosphor layers was suppressed in Penetrons by fabricating a phosphor stack with separation layers. For luminance enhancement, this approach is impossible: we only found a modest increase of the CL by depositing Y_2_O_3_:Eu^3+^ on top of ZnO:Zn due to mixing of the top and bottom layers [19].

### 2.2. Cathodoluminescence in a SEM

**Figure 5:**
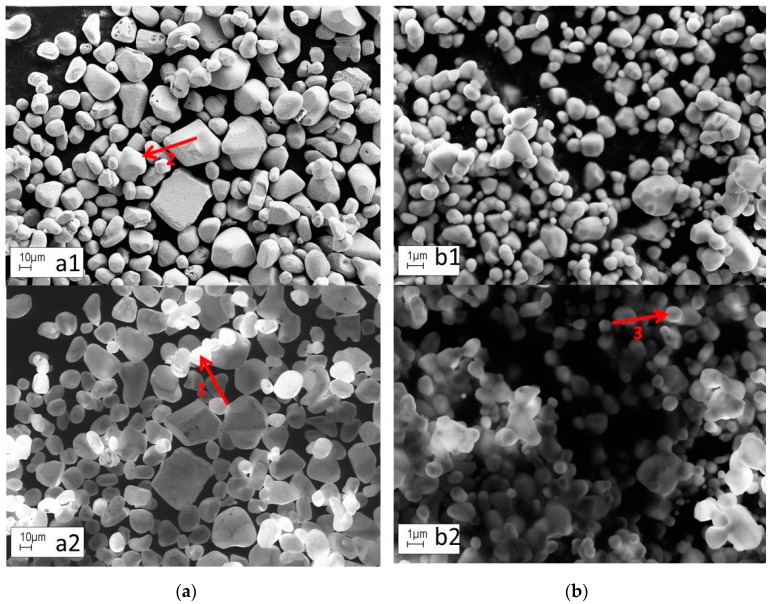
SE- and CL-images of ZnS:Cu,Cl and ZnO:Zn (**b**) at an e-beam voltage of 10 kV: (**a1**) SE-image for ZnS; (**a2**) CL image for ZnS; (**b1**) SE-image for ZnO; and (**b2**) CL-image for ZnO. Substrate is carbon, which has low BS- and SE-coefficients. Same areas are shown in (**a1**) and (**a2**); and in (**b1**) and (**b2**). Please note that the ZnS particles are more than 10 times larger than the ZnO particles.

Although the analysis of CL in a SEM and TEM is a standard technique, we shall describe in this section a simple measuring technique for the decay time of CL in a SEM that we have developed at Brunel University London [20]. Before doing so, we shall briefly introduce CL-microscopy with a SEM. Figure 5 compares SE- and CL-images of ZnS:Cu,Cl and ZnO:Zn particles.

**Figure 6:**
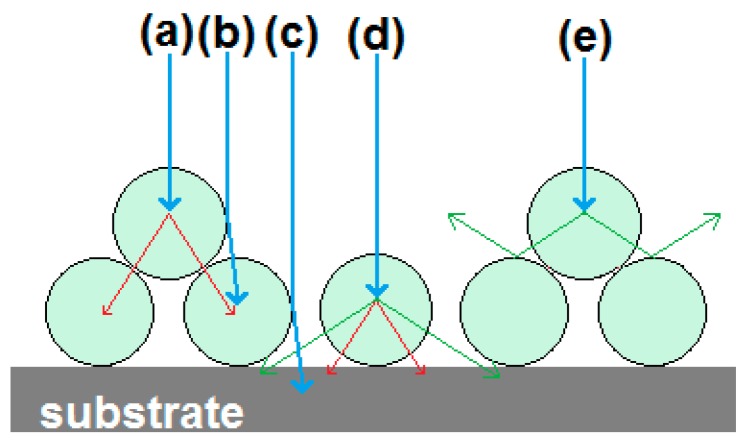
Effect of particle stacking in CL SEM images. Red rays are scattered primary electrons, green CL and blue the electron beam.

Figure 5(a1,b1) shows SE-images, while Figure 5(a2,b2) shows panchromatic CL-images. The SE- and CL-images show different features of the crystals, which helps in analysing these specimens. An eye-catching difference between the SE- and CL-images is the high brightness of some particles on top of others in the CL-images, for example the particles indicated by arrow 1 in Figure 5(a2). This effect is explained in Figure 6. When particles are directly on top of the absorbing substrate, then any primary electrons that penetrate them are lost to the substrate, whereas when they are sitting on other phosphor particles this energy will generate additional CL (cf. rays (a) and (d)). Primary electrons may also be backscattered either onto other phosphor particles as in ray (b) or into the substrate as in ray (c). Additionally, CL that is emitted towards the substrate has a better chance of being scattered to the detector if the particle is located on top of other particles, rather than if it is in direct contact with the carbon-loaded substrate pad (cf. rays (d) and (e)).

In SE-images, one can often observe surface-tilt contrast, which is caused by the reduced penetration depth (and hence enhanced SE-emission) of the electron beam when hitting a tilted surface such as the sides of a phosphor grain, indicated by arrow 2 in Figure 5(a1) [20,28]. CL-images may show contrast enhancement similar to that in SE-images, indicated by arrow 3 in Figure 5(b2). We have explained this contrast enhancement in terms of the ratio between the particle diameter and the electron penetration depth [20].

**Figure 7:**
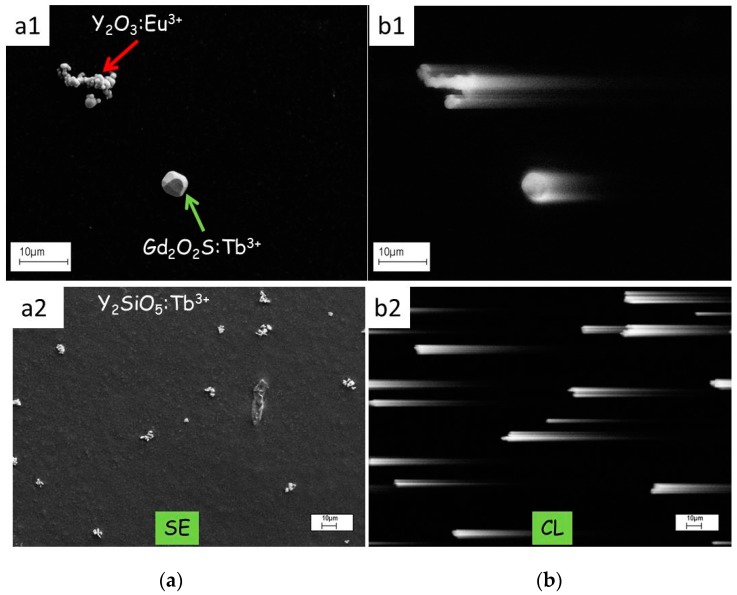
SE- and CL-images of phosphor particles on carbon substrate at 10 kV beam voltage: (**a1**) SE-image of a cluster of Y_2_O_3_:Eu^3+^ particles and one Gd_2_O_2_S:Tb^3+^ particle; (**a2**) SE-image of Y_2_SiO_5_:Tb^3+^ particles; (**b1**) CL-image of same area as (**a1**); and (**b2**) CL-image of same area as (**a2**). The scanning rate for the CL- images was 10.1 s/frame. One frame consisted of (1024 × 768) pixels.

The CL-image of phosphor materials can be smeared out at high scan rates of the SEM, when a particle continues to emit light after the beam has moved onto to a subsequent pixel. Smearing out is detrimental to the picture quality and usually the scan rate is decreased to suppress this effect as much as possible. Smearing out causes comet-like structures, which offer the opportunity to measure the loss of brightness along the tail of these features and, hence to determine the decay time of the materials, as indicated in Figure 7. The images shown in Figure 7 are from reference 20 and have been slightly modified.

**Figure 8:**
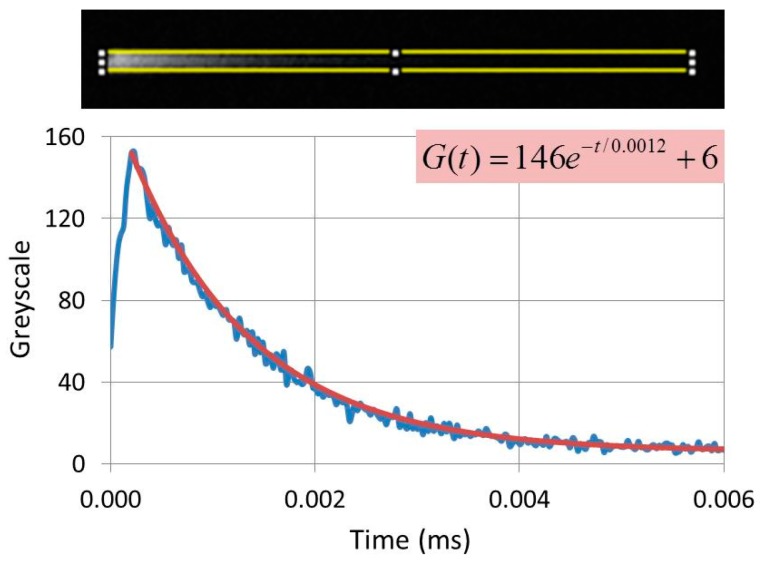
Determination of the decay time of commercial Y_2_O_3_:Eu^3+^ (Nichia, with about 3 mol% Eu^3+^) using ImageJ and curve fitting. Noisy curve is the experimental greyscale, while red curve is the fit. τ_1/e_ is 1.2 ms. Dwell time of the e-beam on the Y_2_O_3_:Eu^3+^ crystal about 250 µs.

The CL-images in Figure 7(b1,b2) show comet-like structures: the length of the tail is a measure of the decay time. The greyscale of the comet was analysed with ImageJ software and the experimental grayscale curve was fitted to an exponential curve *G(t)*, as shown in Figure 8. This curve can generally be written as

(6) $$G(t)=\sum_{i}g_{i}e^{-t/\tau_{i}}+BG$$

where the index *i* labels the transitions that contribute to the light generation; τ*_i_* is the time constant of transition *i*, being the 1/e-value of the decay time (τ_1/e_); t indicates the time; *g_i_* is the maximum value of the exponential *i* at *t* = 0; and *BG* is the background correction. This latter correction depended on the gain setting of the PM tube and was near zero in most cases.

The CL-images shown in Figure 7 are panchromatic, which implies that the decay times determined with this technique are “overall decay times”: these cannot be compared to spectral selective decay times, as indicated in Equation (6). In the case of Figure 8 the emitted light is mainly from the ^5^D_0_→^7^F_2_ (C_2_) transition of Eu^3+^ at 611 nm, because the contribution of other transitions is in the order of a few per cent only and may be neglected. However, if the concentration of Eu^3+^ in Y_2_O_3_ is lowered to 1% and the dwell time of the e-beam on the phosphor particle is shortened, the Eu^3+ 5^D_0_→^7^F_2_ (C_2_) transition gets much more saturated than the ^5^D_1_→^7^F_1_ (C_2_) transition at 533 nm [26]. In that case the grey curve *G(t)* can be represented satisfactorily by two exponentials and by curve fitting we were able to determine the decay times of both transitions [20]. However, in many cases, the grey curves *G(t)* can be represented by a single exponential by optimizing the scanning conditions, as in the case of Figure 8 and the examples that have been summarized in Table 1.

Deviations of *G(t)* from exponential decay may also be an indication of the presence of trapping defects that slow the release of energy into the matrix.

It can be concluded that the technique described above is well-suited for measuring overall decay times of phosphors in the range between 1 μs and 0.1 s; this range is mainly limited by the scan rate of the SEM. This measuring technique is attractive, since it allows the determination of decay times of individual phosphor particles, which may have micrometre or nanometre dimensions [20]. Finally, no additional investments are needed to make these measurements with a (FE)SEM that is equipped with a panchromatic CL-detector such as a PM tube.

### 2.3. Cathodoluminescence Analysis in a TEM

**Figure 9:**
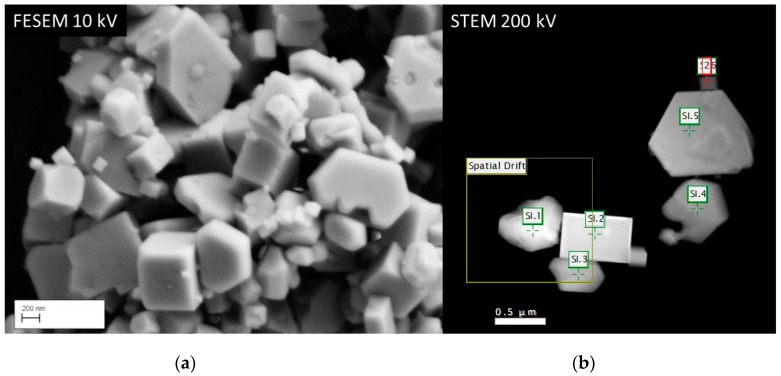
Images of (Lu_0.5_Gd_0.5_)_2_O_2_S:2%Tb^3+^: (**a**): FESEM image at 10 kV; and (**b**) STEM (HAADF) image at 200 kV. SI.1, SI.2, etc., indicate the positions where spectra have been recorded, which are represented in Figure 10b. The Spatial Drift square indicates the part of the image that was used to correct for drift when recording the CL spectra.

In this section we shall present some work that we have recently carried out in the TEM facility of Brunel University London. The main characteristics of this TEM are described in the next section. The work studied the synthesis and luminescence of nanosized (Lu_1-*x*_Gd*_x_*)_2_O_2_S:Tb^3+^ phosphors between *x* = 0 and *x* = 1 with 0.1 and 2 mol% Tb^3+^. This work will be published in total elsewhere [29]; however, an analysis of one sample in the TEM will be presented here. The concentration of Gd^3+^ in (Lu_1-*x*_Gd*_x_*)_2_O_2_S:Tb^3+^ was varied in steps of 0.1 (mol ratio Gd^3+^). We found that during annealing of the hydroxy carbonate precursor with sulphur at 900 °C that the sulphurisation reaction was incomplete for the samples with *x* < 0.7: those samples contained a mixture of Lu_1-*x*_Gd*_x_*)_2_O_3_:Tb^3+^ and (Lu_1-*x*_Gd*_x_*)_2_O_2_S:Tb^3+^, while the sample at *x* = 0 was pure Lu_2_O_3_:Tb^3+^. The hydroxy carbonate precursor was made by homogeneous precipitation with urea in aqueous solutions [30]. From XRD-analyses the concentration of the oxide and oxysulphide phases in the various samples could be determined, because the oxides were cubic crystals and the oxysulphide phase was hexagonal. SEM and STEM pictures of (Lu_0.5_Gd_0.5_)_2_O_2_S:2%Tb^3+^ are shown in Figure 9.

It can be seen in Figure 9 that the range of particle sizes was considerable, from about 100 nm to about 1.5 µm. The drift corrector square, which is indicated in Figure 9, delivers the correction signals to prevent the sample from drifting during recording of the CL-spectra; this feature guarantees that the spectra are recorded exactly at the desired positions. The concentration of oxide phase in this 50/50 sample was found to be 52%. Figure 9a,b shows two types of crystals: cuboid crystals and particles with angles of 120°. It is tempting to assign the cuboid particles to the oxide phase and the other type to the hexagonal oxysulphide.

**Figure 10:**
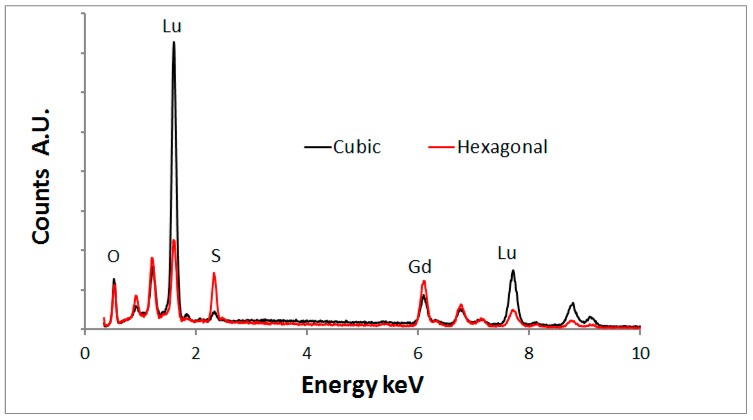
Overlay of EDS-spectra of the cubic and hexagonal type crystals in (Lu_0.5_Gd_0.5_)_2_O_2_S:2%Tb^3+^.

To verify this assignment, we analysed the (Lu_0.5_Gd_0.5_)_2_O_2_S:2%Tb^3+^ sample with energy dispersive X-ray spectroscopy (EDS) in a FESEM and recorded CL spectra of the crystals that are denoted by SI.1, SI.2 etc. in Figure 9b. Figure 10 shows an overlay of the EDS- spectra of the cuboid and hexagonal types.

Figure 10 indicates that the hexagonal type crystals contain much more sulphur than the cubic crystals, which proves that the hexagonal crystals are oxysulphide. A small sulphur signal was detected from the cubic crystals. It is unlikely that this is a part of the crystal structure and it is assumed that the surface of these cubic crystals is contaminated with sulphur. Figure 10 shows also that the lutetium signal is substantially higher than the gadolinium signal for the oxide crystals, whereas the oxysulphide crystals show the opposite. This phenomenon can be explained in terms of segregation of lutetium and gadolinium during the annealing with sulphur. A discussion of this effect is beyond the scope of this article; it will be described in a forthcoming publication [29].

**Figure 11:**
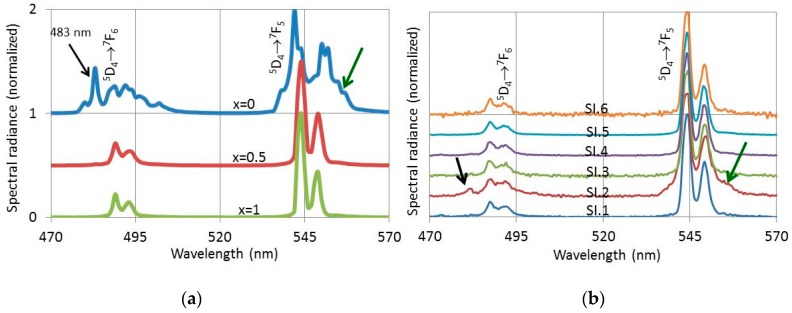
Spectra of (Lu_1-*x*_Gd*_x_*)_2_O_2_S:2%Tb^3+^ between 470 nm and 570 nm: (**a**) PL spectra (excited at 291 nm) of phosphors with *x*_Gd_ = 0, 0.5 and 1; and (**b**) CL spectra of the (Lu_0.5_Gd_0.5_)_2_O_2_S:2%Tb^3+^ crystals shown in Figure 9b excited at 200 keV and room temperature in the TEM.

These CL-spectra that were recorded of the crystals shown in Figure 9b were compared with photoluminescence (PL) spectra that were recorded separately from the powder samples. This comparison is shown in Figure 11.

Figure 11 presents PL- and CL-spectra of (Lu_1-*x*_Gd*_x_*)_2_O_2_S:2%Tb^3+^ between 470 nm and 570 nm. The PL spectra for *x* = 0, 0.5 and 1 in Figure 11a were excited with 291 nm UV light, while the CL-spectra in Figure 11b were recorded with the Gatan spectrometer in the TEM at 200 keV at the various spots indicated in Figure 9b. The spectra in Figure 11 have been normalized towards the maximum peak of the Tb^3+ 5^D_4_→^7^F_5_ manifold at 544 nm and 542 nm for the *x* = 0 spectrum in Figure 11a. This approach facilitated the visualisation of the difference between the oxide spectrum for 100% Lu (*x* = 0) and the oxysulphide spectra (*x* = 0.5 and *x* = 1). It should be realized that the PL-efficiency of the oxysulphide is about 18 times larger than that of the oxide. From the CL-spectra represented in Figure 11b it can be concluded that the spectrum SI.2 has a clear oxide character, because it has an emission peak at 483 nm and the Tb^3+ 5^D_4_→^7^F_5_ manifold at 545 nm is clearly broadened. These are the distinguishing features of the pure oxide spectrum at *x* = 0 in Figure 11a. Because of the large difference in efficiency between oxide and oxysulphide, we conclude that crystal SI.2 is a pure oxide, although the CL spectrum recorded at SI.2 also shows oxysulphide characteristics. The oxysulphide characteristics of the SI.2-spectrum in Figure 11b are due to X-ray excitation of the neighbouring oxysulphide crystals. These X-rays are generated by the beam at SI.2 and cannot easily be eliminated.

Both EDS and CL spectroscopy indicate clearly that the cuboid crystals in Figure 9 are an oxide phase, whereas the hexagonal type crystals are oxysulphide.

## 3. Materials, Methods and Equipment

Y_2_O_3_ phosphors doped with Eu^3+^ or Tb^3+^ and (Lu_1-*x*_Gd*_x_*)_2_O_2_S:Tb^3+^ were made in our laboratory by the urea precipitation method [18,30] followed by annealing at 1020 °C in air for the oxides or at 900 °C with S for the oxysulphides [31]. All other phosphor materials were bought from commercial suppliers and used without additional purification [18,19,20].

Phosphor powder layers were deposited onto the ITO-coated glass slides by settling from isopropanol suspensions containing various phosphor concentrations for the CL-efficiency measurements. These suspensions were dispersed by ultrasonic cavitation prior to settling. Coating weights were determined by weighing the slides. The slides were mounted in holders that also contained the shield and grid construction to deflect SEs [18,19]. These holders were fixed on manipulating rods for translational or rotational displacements in the vacuum chambers; sample plate, reference plate and shield were electrically connected to external power supplies and ammeters.

For the SEM analyses the phosphor powders were deposited onto carbon pads from diluted suspensions in water or isopropanol. In some cases the phosphor powder was dusted onto the sticky carbon pad. For the studies in the TEM copper grids coated with holey carbon films were used as substrates: these are transparent to the high-energy electrons.

The CL measurements for luminous efficiency were carried out in two different high vacuum chambers at a vacuum level of 3 × 10^−6^ mbar using Kimball Physics Inc., USA, electron guns and associated power supplies over the ranges of electron beam voltages of 1–5 kV and 2–15 kV respectively. The 2–15 kV rig has schematically been depicted in Figure 1. The electron guns had the ability to focus and defocus the beam over a range of beam diameters, while the grid of the Wehnelt triode enabled the adjustment of currents between 0 and about 25 µA. For the studies of the luminous efficacy a uniform electron beam (by defocusing) and a current density of 1 μA/cm^2^ was used. Deflection plates enabled optimum positioning of the electron beam on the sample and the ZnO:Zn reference. The latter being a non-charging thin film of ZnO:Zn powder on ITO to adjust the current on the charging Y_2_O_3_:Eu^3+^ samples, as explained in Section 2.1. For the determination of the luminous efficacy and energy efficiency, luminance and spectral radiance, respectively, were recorded with two Spectrobos 1200 spectroradiometers manufactured by JETI, Germany, between 380 and 780 nm in reflection and transmission mode [17,18]. High resolution spectra (±0.2 nm) were also recorded with a Bentham, UK, monochromator detector system between 350 and 800 nm.

**Figure 12:**
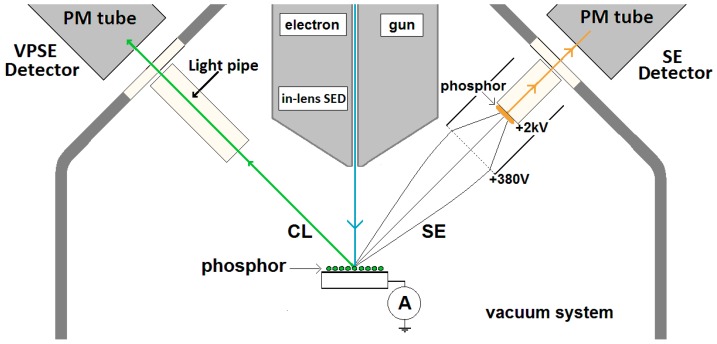
Schematic of the Zeiss Supra 35VP FESEM capable of recording SE- and CL-images. Detector systems are explained in the text.

Figure 12 shows schematically the geometry of the Zeiss Supra 35VP field emission scanning electron microscope (FESEM) used in this work. The system is equipped with four detector systems. The first is an Everhart–Thornley (ET) SE-detector, which collects primarily SEs, although some BSEs may also contribute. There is also an in-lens SE-detector, for use when a very short working distance is required, and this detects only SEs. The BSE detector has not indicated in Figure 12.

The microscope has the possibility to operate in high pressures (<133 Pa) to facilitate imaging of specimens that charge under the beam. Since it is impossible to operate the ET SE-detector at high pressures, an additional detector has been installed. This operates by using a PM-tube to detect the fluorescence generated when low-energy SEs, emitted from the surface (under bombardment from the primary electron beam), excite the gas (nitrogen) in the chamber. If this detector is used under high vacuum conditions, then the N_2_ fluorescence is absent and the PM-tube is capable of generating high quality CL-images from luminescent materials.

The Cl-images produced in the Zeiss SEM are panchromatic. The response time of the PM tube is in the nanoseconds range; so, its effect on decay times in the micro- and milliseconds range can be neglected. Image analysis of the panchromatic CL-micrographs was performed for the determination of the decay times using ImageJ (Public Domain) software. The elemental composition of individual particles was studied by EDS using an EDAX (part of the Ametek Inc. groups, Berwyn, PA, USA) instrument fitted with an Octane Super lithium-drifted silicon detector.

Phosphor samples were also investigated with a TEM, model 2100F, JEOL, Japan, equipped with a Schottky-type field emission gun. The spot size of the e-beam in the scanning mode (STEM) at the specimen was adjusted to 0.2 nm or 1.5 nm. Initial work demonstrated the need to reduce the X-rays in the column generated from the condenser lens aperture, which were found to significantly contribute to disperse excitation of phosphor samples. These X-rays excited the phosphor and caused the emission of visible light when the electron beam was not on the sample, leading to unwanted interference and a loss of resolution. To reduce this X-ray excitation of the sample, a hard X-ray aperture was inserted into the column, which reduced the background noise in CL imaging and spectroscopy modes considerably. The TEM was equipped with a Vulcan™ CL detector, Gatan, USA, for imaging and spectroscopic purposes. This system used a Czerny-Turner spectrometer with back-illuminated CCD and a grating with 1200 lines/mm (blazed at 500nm) for collection of CL emission spectra. Light was collected from the sample using a mirror above and below the sample, which enabled a solid angle of about 5 sr, which is almost half of a sphere. This high solid angle made light collection highly efficient and enabled the collection of CL at low intensity. By collecting the CL with the Vulcan system simultaneously with JEOL’s high-angle annular dark-field (HAADF) detector, it was possible to record CL-spectra from individual nanocrystals. A small cryostat connected to the sample holder enabled cooling of the samples in the TEM down to 102 K (−171 °C); adjustment of the sample temperature anywhere between 102 K and 303 K was facile.

## 4. Conclusions

In the previous sections, we have described three CL-spectroscopy techniques that have recently been developed in Brunel University London. The description of the comparison method for the determination of CL-efficiency is the most elaborate, because we thought it to be useful in view of the large variation of efficiency values in the literature. With the comparison method it must be possible to reduce this variation. A round robin procedure with carefully selected samples and participating laboratories might prove this expectation. The determination of the decay time with an SEM is straightforward when the SEM has been equipped with a CL-detector. Depending on the scan rates of the SEM, decay times between 2 µs and 0.1 s can be determined. Finally, we described how Brunel’s TEM enables assignments of crystals in a mixture by CL-spectroscopy.

## Figures and Tables

**Table 1:** Decay times determined from CL-images in Figure 7 and Figure 8.

Material Source	τ_1/e_ (ms)		Ref.
	This Work	Literature	
Y_2_O_3_:Eu^3+^ (Nichia)	1.2	1.2, 1.12, 1.1	[20]
Y_2_O_3_:Eu^3+^ (Brunel)	1.0	1.06	[20,26]
Y_2_SiO_5_:Tb^3+^ (Nichia)	2.8	3.2	[20]
Gd_2_O_2_S:Tb^3+^ (Nichia)	0.56	0.558	[20]
